# Impact of *TP53* mutations in breast cancer: Clinicopathological features and prognosisImpact of *TP53* mutations in breast CA

**DOI:** 10.1111/1759-7714.13467

**Published:** 2020-05-15

**Authors:** Xuerui Li, Xiaoqing Chen, Lingzhu Wen, Yulei Wang, Bo Chen, Yunlian Xue, Liping Guo, Ning Liao

**Affiliations:** ^1^ Department of Breast Cancer, Cancer Center, Guangdong Provincial People's Hospital Guangdong Academy of Medical Sciences Guangzhou China; ^2^ The Second School of Clinical Medicine Southern Medical University Guangzhou China; ^3^ Statistics Office, Information and Statistics Center, Guangdong Provincial People's Hospital Guangdong Academy of Medical Sciences Guangzhou China

**Keywords:** Breast cancer, genomic mutation, next‐generation sequencing, *TP53*

## Abstract

**Background:**

*TP53* is a crucial tumor suppressor gene. However, the mutation pattern of *TP53* in Chinese patients with breast cancer has not yet been determined.

**Methods:**

A total of 411 untreated patients with invasive breast cancer diagnosed at Guangdong Provincial People's Hospital (GDPH) between June 2017 to September 2018 were recruited into the study. Mutational alterations in *TP53* were detected and correlations between *TP53* mutations and clinicopathological features analyzed. Comparative analysis of the data in the GDPH cohort with those in the METABRIC cohort were carried out.

**Results:**

A significantly higher rate of *TP53* mutations was detected in the GDPH cohort (51.3%) compared with the METABRIC cohort (34.4%) (*P* < 0.01). In the GDPH cohort, 77.8% of the mutations were located in the conserved areas across exons 5–8 of *TP53*; among these, 112 were identified as missense mutations and mainly clustered in the DNA‐binding region. R273C/H (*n* = 11) and R248Q/W (*n* = 10) were two of the most common mutation sites of *TP53* detected in the cohort of GDPH patients. Logistic regression multivariate analysis showed that histological grade III, ki‐67 > = 25%, HR‐ and Her2+ in breast cancer had higher mutation probability of *TP53* (*P* < 0.001 in the GDPH cohort). Furthermore, receiver operating characteristic (ROC) model combining molecular typing and Ki‐67 was established to predict the mutation of *TP53*, and the AUC was 0.846.

**Conclusions:**

A significantly higher rate of *TP53* mutation was detected in the Chinese cohort compared with the METABRIC. Correlation analysis revealed a significant association of *TP53* mutation with HR‐ and HER2+, higher Ki‐67 and histological grade in breast cancer patients.

## Introduction

Breast cancer is the most prevailing cancer in women worldwide^1^; its pathogenesis involves multiple risk factors as well as a hormone‐dependent process. Extensive efforts have been made on sequencing the whole genome of the tumor cells of breast cancer, reiterating the genomic heterogeneity of this complex disease.^2^ Combinatorial application of powerful high‐throughput screening techniques with advanced methods and protocols in bioinformatics has greatly facilitated the understanding of molecular mechanisms underlying the carcinogenesis.^3^, ^4^ Notably, immunohistochemistry‐based studies usually cause an excess number of misdiagnosed cases and increase the interstudy discrepancy. Nowadays, gene expression patterns are emerging as important indexes in the decision‐making of cancer treatments.

*TP53* was originally identified in the 1970s as a viral SV40 T antigen interacting protein and has been shown to function as a tumor suppressor.^5^ It has been reported that almost all types of cancers harbor somatic TP53 mutations with varied rates ranging from 50% to 5%.^6^, ^7^ Previous studies have suggested that *TP53* status is crucial for the response of cancer patients to multiple anticancer therapies. In addition, *TP53* mutations may be causally linked to the drug resistance and failed treatment,^8^ and are therefore closely related to poor prognosis in multiple cancer types.^9^ In the case of breast cancer, significance of *TP53* mutations in the prognosis or drug response prediction has been assessed in over 20 studies.^10^ However, it remains unclear whether *TP53* functions as an independent prognostic factor.

Although the incidence of breast cancer in Asia is relatively lower than that in western countries, it has been rising progressively in China during the past few decades and may eventually surpass that in the West. It has been shown that breast cancer displays a considerable variation in both clinicopathological characteristics and prognosis from one racial and ethnic group to another.^11^, ^12^, ^13^ Unfortunately, the understanding and management of breast cancer have been mainly dependent on research and data from the West. Accordingly, there is still a pressing need to determine whether TP53 mutations are associated with clinicopathological features of breast cancer in the Chinese population.

The current study performed next‐generation sequencing technique (NGS) to analyze the mutational profile in a large Chinese cohort from GDPH and compared the findings with the METABRIC data. This preliminary study characterized the molecular and clinical significance of TP53 mutations detected in the patients, revealing a marked difference in the molecular pattern between the GDPH and METABRIC cohorts.

## Methods

### Patient selection and sample collection

This project obtained the ethical approval from GDPH and conformed to the Helsinki Declaration.^14^ The GDPH cohort was comprised of 411 female breast cancer patients collected at the hospital from June 2017 to September 2018. All recruited patients met the following criteria: (i) A definite diagnosis as the invasive cancer; (ii) available sequencing data on primary tumor tissue qualified for this study; (iii) a full set of data regarding the clinicopathological characteristics; and (iv) a full set of clinical data including gender, onset age, menstrual state, primary tumor dimension, axillary lymph node metastasis, distant metastasis, pathological classification, histological grade, molecular type, estrogen receptor (ER), progesterone receptor (PR), human epidermal growth factor receptor 2 (HER2), proliferating nuclear antigen Ki‐67,as well as TNM staging. Tissue samples from the primary tumors were obtained and detected by the sequencing.

We selected 1985 patients with detail clinical data from 2509 patients in the METABRIC database for comparative analysis in this study. The data of all cases with respect to *TP53* mutation patterns as well as clinicopathological parameters are available online (http://www.cbioportal.org).

### Extraction of DNA

Formalin‐fixed paraffin‐embedded (FFPE) was conducted using the tissue kit (QIAGEN, California, USA)based on the recommended protocol. Extraction of genomic DNA was performed by using QIAamp and Qubit dsDNA assay was used to determine the DNA concentration.

### Preparation of DNA library and NGS

In this study, the sequencing was performed on collected tissue samples from the primary tumor. The whole procedure from the sample preparation and NGS to data analysis was conducted as previously described.^15^

### Statistical analysis

The statistical analysis was performed using SPSS 22.0 software (IBM). The frequency and percentage were used in data collection for calculating categorical variables. Intergroup differences were analyzed using Fisher's exact or Chi‐square test. Kruskal‐Wallis test on different variables was performed for determining the correlations between them. The correlations between *TP53* mutation and clinicopathological features was analyzed by using logistics regression and receiver operating characteristic (ROC) curve data in this study were indicated as mean ± SD. *P* < 0.05 was considered statistically significant.

## Results

### High frequency of *TP53* mutations in breast cancer

To characterize the functional relevance of *TP53* mutations in breast cancer, we analyzed the mutation frequency of *TP53* detected in METABRIC breast cancer samples. As shown in cBioPortal (www.cbioportal.org), *TP53* mutations were detected in 863 of 2509(34.4%) cases, designating *TP53* as one of the most frequently mutated genes in this type of cancer. In our previous study, we reported that the rate of *TP53* mutations were detected in 137 of 304 (45%) cases.^16^ In this study, we expanded the sample size to 411 cases and found that the rate of *TP53* mutations were 51.3%, significantly higher than that of METABRIC. These data suggest that *TP53* is implicated into breast cancer pathogenesis.

### Distinct mutational spectra of *TP53* in the two different cohorts

NGS was performed to screen *TP53* mutations in the cohort of GDPH breast cancer patients. Somatic *TP53* mutations were detected in 211 out of 411 (51.3%) patients in this cohort. Consistent with previous reports,^2^, ^17^ we observed that the *TP53* mutations were unevenly distributed within the entire gene. Notably, 77.8% of the mutations (164/211) detected in the cohort located in the conserved areas across exons 5–8; among the 164 mutations, 112 were found to be missense mutations and mainly clustered within the DNA‐binding region of TP53. By contrast, the remaining *TP53* mutations (47/211, 22.2%) were detected in the coding regions beyond exons 5–8; a large portion of these mutations (39/47) were identified as nonmissense ones containing 20 indels (in‐frame and frameshift) and 11 nonsense mutations, as well as eight splicing variants. As summarized in Table 1, total 27 codons of TP53 were found to be frequently mutated in the cohort of GDHP patients.

**Table 1:** Prevailing mutations of *TP53* detected in the GDPH cohort (≥2 altered cases in our cohort)

Position (codon)	Protein/amino acid change	Exon	Type of mutation	No. of mutated cases
93	p.L93fs	4	Frameshift	2
107	p.Y107*	4	Nonsense	2
151	p.P151S	5	Missense	2
163	p.Y163C	5	Missense	3
173	p.V173L	5	Missense	2
175	p.R175H	5	Missense	6
176	p.C176F	5	Missense	2
179	p.H179R	5	Missense	3
183	p.S183*	5	Nonsense	2
192	p.Q192*	6	Nonsense	7
194	p.L194R	6	Missense	2
218	p.V218fs	6	Frameshift	2
213	p.R213*	6	Nonsense	2
220	p.Y220C	6	Missense	2
238	p.C238F	7	Missense	2
248	p.R248Q	7	Missense	7
248	p.R248W	7	Missense	3
273	p.R273C	8	Missense	6
273	p.R273H	8	Missense	5
275	p.C275Y	8	Missense	2
278	p.P278R	8	Missense	2
280	p.R280fs	8	Frameshift	2
282	p.R282W	8	Missense	2
307	p.A307V	9	Missense	2
331	p.Q331*	9	Nonsense	2
342	p.R342*	10	Nonsense	4
342	p.R342fs	10	Frameshift	3

* Indicates the nonsense mutation.

**Figure 1:**
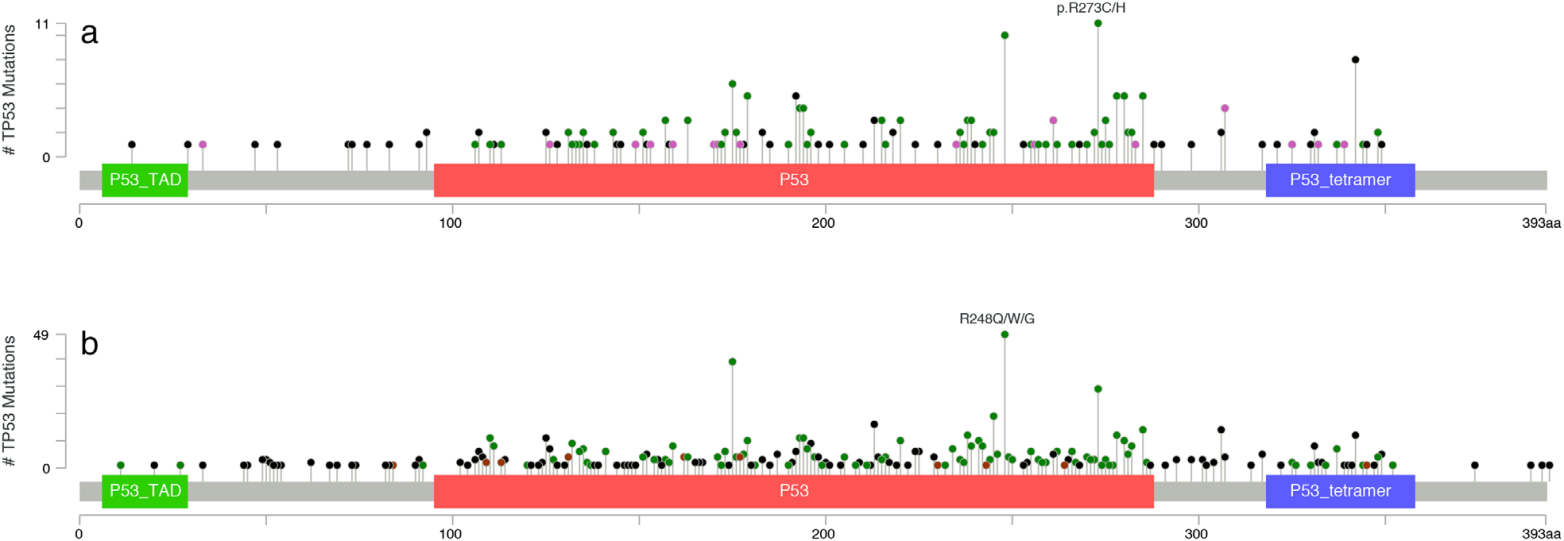
(**a**) The mutational spectra of *TP53* in GDPH cohort; (**b**) METABRIC cohort (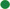) Missense (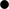) Trucating (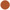) Inframe (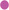) other.

Next, we performed comparative analysis of the *TP53* mutation spectra between the GDPH and METABRIC cohorts (Fig 1a,b. Despite the highly similar distributions of *TP53* mutations identified in these two cohorts, a distinct difference in the mutational hotspots between them was present. In this case, we identified R273C/H (*n* = 11) and R248Q/W (*n* = 10) located at *TP53* DNA‐binding domain as the two of the most common mutation sites in the GDPH cohort. By contrast, R248Q/8W/8G/9mfs*19 (*n* = 60), R175H/G/Lif*5 (*n* = 42) and R273H/C/L/G/P/V274_R282del (*n* = 39) was found to be the three of the most common mutation sites detected in the METABRIC cohort, whereas we only detected six cases of R175H mutations in the GDPH patients. Moreover, we identified codons 192 (*n* = 7) and 342 (*n* = 7) as mutational hotspot‐harboring codons of *TP53* in the patients that were yet to be previously reported.^18^

### Correlation analysis of *TP53* mutations with clinicopathological characteristics

To characterize the clinicopathological relevance of *TP53* mutations in breast cancer, we first divided all selected patients in each of the two cohorts from databases into wild‐type and mutant *TP53* groups, and then analyzed the correlations between *TP53* mutations and clinicopathological parameters.

**Figure 2:**
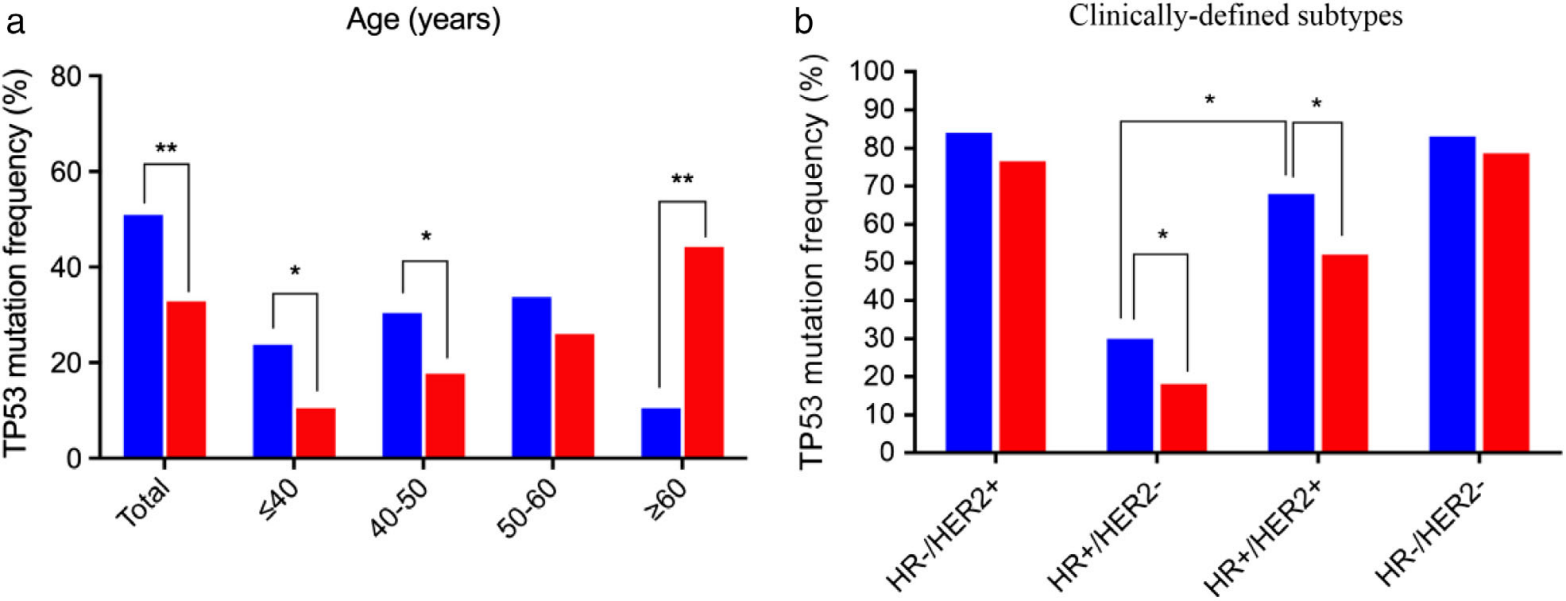
(**a**) Classification of *TP53* mutations by the onset age of patients (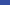) GDPH and (**b**) clinically‐defined subtypes (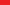) METABRIC.

Figure 2a indicated the percentage of *TP53* mutation positive patients in each of different age groups. Although the age distribution of patients with *TP53* mutation in GDPH and METABRIC data was significantly different, the multivariate analysis of the two groups of data indicated that age was not correlated with the mutation of *TP53*. As shown in Fig 2b, the proportion of *TP53* mutations in different HR/HER2 status was different between the two groups.

Logistic regression single factor analysis showed that tumor stage, pathological grade, HR/HER2 status and ki‐67 may be related to *TP53* mutations in the GDPH cohort. Multivariate analysis of these factors revealed that only pathological grade, HR/HER2 status and ki‐67 were independent influencing factors for *TP53* mutation. Among them, *TP53* mutation carriers were significantly more likely to identified as pathological grade 3, ER‐, PR‐, HER2+ and ki‐67 > = 25% (*P* < 0.01). However, ki‐67 data was not provided in the METABRIC cohort and the statistical analysis suggested that the effect of pathological grade and HR/HER2 status on *TP53* mutation was consistent with GDPH (*P* < 0.01), and there was no correlation between tumor stage and mutation of *TP53* (*P* > 0.05).

**Figure 3:**
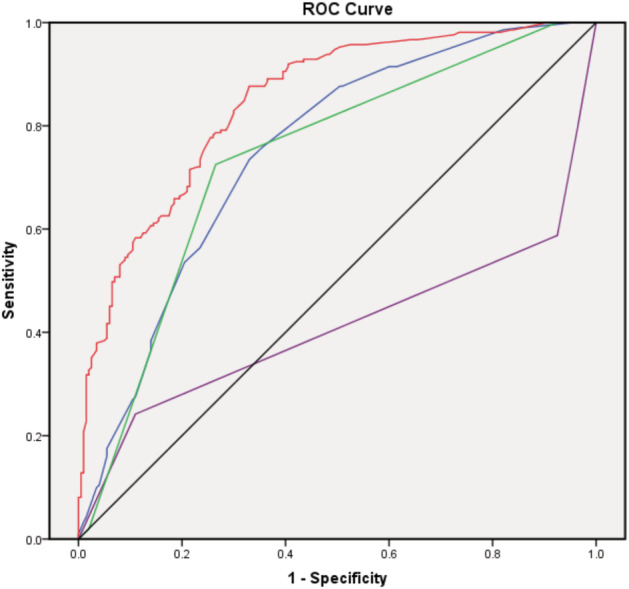
ROC curve in GDPH cohort (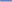) Ki‐67 (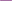) HR/HER2 typing (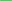) Histological_grade (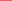) Predicted .probability (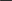) Reference Line.

The correlation analysis of TP53 mutations with clinicopathological parameters is summarized in Table 2. We used multivariate logistic regression techniques to perform the receiver operating characteristic (ROC) analysis in GDPH cohort, and found that the combined pathological grade, ki‐67 and HR/HER2 status could predict the mutation of TP53, and the AUC was 0.846 (Fig 3, Table 3), and the cutoff setting of ki‐67 was 25%.

**Table 2:** Clinicopathological parameters of the patients in the two cohorts

	METABRIC Cohort			GDPH Cohort		
	WT TP53	MT TP53	*P*	WT TP53	MT TP53	*P*
	*n* = 1325	*n* = 660		*n* = 200	*n* = 211	
Variable	No. (%)	No. (%)		No. (%)	No. (%)	
Age (year)			0.052			0.139
≤60	509(58.1)	367(41.9)		162 (46.0)	190 (54.0)	
>60	816(73.6)	293(26.4)		38 (64.4)	21(35.6)	
Histologic grade			<0.001			<0.001
I	162 (95.9)	7 (4.1)		15 (100)	0 (0.0)	
II	639 (82.7)	134 (17.3)		132 (69.1)	59 (30.9)	
III	448 (46.9)	507 (53.1)		49 (24.7)	149 (75.3)	
NA	76 (86.4)	12 (13.6)		4 (50.0)	4 (50.0)	
ER status			<0.001			<0.001
Positive	1207 (79.9)	304 (20.1)		183 (62.5)	110 (37.5)	
Negative	118 (24.9)	356 (75.1)		17 (14.4)	101 (85.6)	
PR status			<0.001			<0.001
Positive	858 (82.5)	182 (17.5)		172 (63.2)	100 (36.8)	
Negative	463 (49.3)	477 (50.1)		21 (15.9)	111 (84.1)	
NA	4 (80.0)	1 (20.0)		0 (0.0)	0 (0.0)	
HER2 status			<0.001			<0.001
Positive	86 (35.1)	161 (64.9)		25 (21.4)	92 (78.6)	
Negative	1235 (71.3)	498 (28.7)		161(59.2)	111(40.8.6)	
Equivocal and NA	4 (80.0)	1 (20.0)		14(63.6)	8 (36.4)	
Ki67			NA			<0.018
<25	NA	NA		129 (71.7)	51 (28.3)	
≥25	NA	NA		71 (30.7)	160 (69.3)	
HR/HER2 typing			<0.001			<0.001
HR+/HER2‐	1164 (80.9)	274 (19.1)		163 (69.1)	73 (30.9)	
HR+/HER2+	56 (45.2)	68 (54.8)		22 (30.1)	51 (69.9)	
HR‐/HER2+	26 (22.4)	90 (77.6)		7 (14.3)	42 (85.7)	
HR‐/HER2‐	56 (20.3)	220 (79.7)		8 (15.9)	45 (84.1)	
NA	23 (74.2)	8 (15.8)		0 (0.0)	0 (0.0)	
pTNM stage			0.177			0.074
0	10 (83.3)	2 (16.7)		0 (0.0)	0 (0.0)	
I	366 (73.1)	135 (26.9)		61 (59.8)	41 (40.2)	
II	548 (66.1)	281 (33.9)		93 (48.6)	98 (51.4)	
III	61(51.7)	57 (48.3)		36 (39.6)	55 (60.4)	
IV	8 (80.0)	2 (20.0)		10 (38.5)	16 (61.5)	
NA	332 (64.5)	183 (35.5)		0 (0.0)	1 (100)	

ER, estrogen receptor; PR, progesterone receptor; HR, hormone receptor; MT, mutant; WT, wild‐type; NA, not applicable.

**Table 3:** Area under the curve(AUC)

				Asymptotic 95% confidence interval	
Test result in variable	Area	Standard error^†^	Gradual Sig.^‡^	Lower limit	Upper limit
Ki‐67	0.748	0.024	0.000	0.701	0.796
HR/HER2 typing	0.411	0.029	0.002	0.353	0.468
Histological grade	0.736	0.025	0.000	0.686	0.785
Prediction probability	0.846	0.019	0.000	0.809	0.883

Test result in variable: Ki‐67, HR/HER2 typing, Histological grade, Prediction probability.

† Under the nonparametric hypothesis.

‡ Null hypothesis: real area = 0.5.

### Clinical impacts of *TP53* mutations

**Figure 4:**
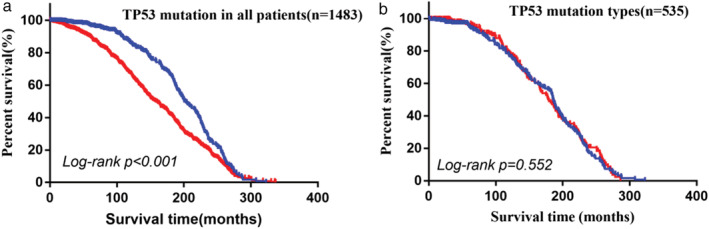
Kaplan‐Meier survival curves indicating OS associated with varied TP53 status in the METABRIC cohort (**a**) (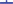) WT TP53 (*n* = 948), and the cases grouped based on the mutation types (**b**) (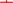) MT TP53 (*n* = 535).

As depicted in Fig 4a,b, *TP53* mutations were correlated with a poor clinical outcome in METABRIC cohort (HR, 1.56; 95% CI: 1.35–1.80, *P* < 0.01), whereas there was no significant correlation between the mutation types and clinical outcome (HR, 1.02; 95% CI, 0.80–1.31, *P* > 0.05). The data indicated that these mutations have the same prognostic significance as those located in the DNA‐binding region.

## Discussion

In the present study, we aimed to delineate the mutation rates and characteristics of *TP53* through NGS in a large cohort comprising 411 Chinese breast cancer patients and compare the obtained data in a METABRIC cohort. Consistent with previous observations,^19^ we found that mutations detected in Chinese patients were mostly clustered into the DNA binding region of TP53 (exons 5–8). Notably, nearly 20% of the mutations detected in this cohort were identified in the coding region beyond exons 5–8. Previous studies suggested an association of *TP53* mutations with the prognostic status of the cancer patients.^19^, ^20^ In particular, compared with the missense mutations of *TP53*, the nonmissense mutations displayed a more strong association with poor prognosis in the patients.^18^ On the contrary, in the present study, we did not observe a significant difference on the prognostic impact of *TP53* mutations among the different mutation types. Furthermore, the prognostic status associated with the above mutations was not significantly different from that linked to those mutations located in the DNA‐binding region (*P* > 0.05). Overall, DNA sequencing‐based mutation screening of all *TP53* exons can provide valuable information with respect to clinical assessments of breast cancer patients.

Comparative analysis revealed that a significantly higher rate of *TP53* mutations is detected in the Chinese cohort compared with the METABRIC cohort (51.3% vs. 33.2%; *P* < 0.01). The discrepancy between the two cohorts was justified by the reports that *TP53* mutations were identified in approximately 30%–40% of patients with primary breast cancer in western countries.^4^, ^21^, ^22^, ^23^ It may be related to the proportion of patients in stage III + IV (28.5% in GDPH cohort vs. 6.4% in METABRIC cohort). In addition, we identified R273C/H as the most prevalent *TP53* mutation detected in GDPH cohort, while R248Q/W/G was found to be the most frequent mutation among the tumor suppressor genes in the METABRIC cohort. It has been speculated that the occurrence of mutation hotspots in a given gene can be attributed to combinatorial effects of the greatly mutable sequence context and specific mutation‐caused selective growth advantage.^24^ Despite the presence of a significant difference in the mutational spectra of *TP53* between the two cohorts, it remains to be determined whether the mutation hotspots identified in the GDPH patients are relevant to the treatments for breast cancer.

In the meantime, correlation analysis showed that an association of *TP53* mutations with a subset of clinicopathological parameters is present in both Chinese and METABRIC patients. Although multiple studies in which a markedly higher rate of *TP53* mutations was detected in young women, as well as medullar carcinoma, suggesting an involvement of *TP53* mutations into the hereditary cancer,^25^, ^26^, ^27^, ^28^, ^29^ the data of GDPH and METABRIC did not indicate that age was an independent factor of *TP53* mutations (*P* > 0.05). It has consistently been reported that a higher rate of *TP53* mutation has been identified in breast cancer patients at advanced stages or with aggressive characteristics, including the subtype of triple negative or HER2 amplified cases.^30^, ^31^ However, our data only supported the effect of HR/HER2 status on *TP53*, but did not find the effect of tumor stage on *TP53*, which may be related to the small number of cases included in the cohort of GDPH stage IV breast cancer patients, or other reasons. In addition, *TP53* mutations have been shown to be linked to elevated global genomic instability as well as enhanced cell proliferation related indicators including high mitotic rate, highly expressed Ki‐67, and highly expressed cyclin E.^32^, ^33^ In our study, factors with poor clinical prognosis such as higher Ki‐67, higher pathological grade, HR‐, HER2+ can predict whether *TP53* has mutation, among which higher Ki‐67 and higher pathological grade have the greatest impact.

Together, these studies identified *TP53* mutations as associated factors of advanced breast cancer with aggressive characteristics.

Obviously, this study was limited by the following points. First, we did not present prognosis related data of those *TP53* mutation positive patients in the GDPH cohort. Second, although this study reported that higher Ki‐67, higher histological grade, HR‐ and HER2+ could predict *TP53* mutations from the GDPH cohort, these clinical high‐risk factors suggest poor prognosis in patients, and the relationship between *TP53* mutation type and prognosis have not as yet been determined. There is no reliable research evidence to prove the relationship between *TP53* mutation type and prognosis, and we need to further explore the mechanism which underlies the role of this tumor suppressor gene in carcinogenesis.

## Disclosure

The authors declare that there are no competing interests
